# Genetic variations of *MUC17* are associated with endometriosis development and related infertility

**DOI:** 10.1186/s12881-015-0209-7

**Published:** 2015-08-19

**Authors:** Ching-Wen Yang, Cherry Yin-Yi Chang, Ming-Tsung Lai, Hui-Wen Chang, Cheng-Chan Lu, Yi Chen, Chih-Mei Chen, Shan-Chih Lee, Pei-Wen Tsai, Su-Han Yang, Chih-Hung Lin, Jim Jinn-Chyuan Sheu, Fuu-Jen Tsai

**Affiliations:** The Institute of Basic Medical Sciences, National Cheng Kung University, Tainan, Taiwan; Institute of Biomedical Sciences, National Sun Yat-sen University, Kaohsiung, Taiwan; Department of Obstetrics and Gynecology, China Medical University, Taichung, Taiwan; Institute of Environmental Health, China Medical University, Taichung, Taiwan; Department of Pathology, Taichung Hospital, Taichung, Taiwan; School of Medicine, China Medical University, Taichung, Taiwan; Department of Pathology, Medical College, National Cheng Kung University, Tainan, Taiwan; Human Genetic Center, China Medical University Hospital, Taichung, Taiwan; Collage of Medical Science and Technology, Chung Shan Medical University, Taichung, Taiwan; Department of Pathology, Kaohsiung University Hospital, Kaohsiung, Taiwan; School of Chinese Medicine, China Medical University, Taichung, Taiwan; Department of Health and Nutrition Biotechnology, Asia University, Taichung, Taiwan; School of Post-Baccalaureate Chinese Medicine, China Medical University, Taichung, Taiwan

## Abstract

**Background:**

Genetic alterations of mucin genes, such as *MUC2* and *MUC4*, were previously identified to be associated with endometriosis and related infertility. Additionally, gene expression profiling has confirmed MUC17 to be overexpressed in mucinous ovarian carcinoma; however, its associated risk for endometriosis remains unclear. This study was focused on the potential impact of genetic variations in *MUC17* on endometriosis development and associated clinical features.

**Methods:**

The study subjects included 189 female Taiwanese patients with pathology-proven endometriosis and 191 healthy Taiwanese women as controls. Five single-nucleotide polymorphisms (rs4729645, rs10953316, rs74974199, rs4729655, and rs4729656) within the *MUC17* gene were selected and genotyped using the *Taqman* genotyping assay to examine the allele frequency and genotype distributions of *MUC17* polymorphisms.

**Results:**

Genotyping revealed that the A allele at rs10953316 in *MUC17* was a protective genetic factor in endometriosis development (*p* = 0.008; OR = 0.53; 95 % CI: 0.36-0.79). Genetic variation of rs4729655 protected patients against endometriosis-induced infertility, but was associated with a higher cancer antigen 125 (CA125) level. Base-pairing analysis, called MaxExpect, predicted an additional loop in the mRNA structure caused by rs10953316 polymorphism, possibly influencing ribosome sliding and translation efficiency. Such predictions were confirmed by immunohistochemistry that patients with AA genotype at rs10953316 showed low MUC17 levels in their endometrium, patients with GA genotype showed moderate levels, and strong staining could be found in patients with GG genotype.

**Conclusions:**

*MUC17* polymorphisms are involved in endometriosis development and the associated infertility in the Taiwanese population.

**Electronic supplementary material:**

The online version of this article (doi:10.1186/s12881-015-0209-7) contains supplementary material, which is available to authorized users.

## Background

Endometriosis is a common chronic gynecological disease described as the presence of endometrial glands and stroma located outside the uterine cavity, ovaries, fallopian tubes, and even on the bladder or intestines [1, 2]. It occurs in approximately 10 % of women or teenage girls at reproductive age. Up to 50 % of patients also suffer from infertility [3, 4]. Clinical symptoms of endometriosis include several types of pain, such as excessive menstrual pain, pelvic pain with defecation, and chronic pelvic pain [5]. Epidemiological studies revealed a higher risk for endometriosis patients to develop different types of ovarian cancers [6, 7]. Molecular pathological analyses have provided strong evidence to support the histological transition from benign endometriosis to ovarian malignancy [8, 9].

Mucins are high-molecular-weight o-glycoproteins that function as the protective and lubricative layers on epithelial surfaces, such as the respiratory, gastrointestinal, and reproductive tracts [10–13]. Gene expression analyses have indicated that MUC1 ~ 4 are the major mucins constitutively expressed in endometrial epithelium, and their levels can be controlled by the menstrual cycle [14, 15]. With potent roles in cellular proliferation, MUC1 and MUC4 overexpression has been identified as a tumor marker for endometriotic lesions and certain types of ovarian tumors [16, 17]. Interestingly, genetic polymorphisms in *MUC2* and *MUC4* were recently reported to be associated with endometriosis development and the related infertility [18, 19]. Because endometriosis shares several similarities with cancer (e.g., highly mobile and proliferative) these data support that alterations in mucins either at the expression level or within their genetic sequences may have a significant impact on endometrium regulation and development.

In this study, we investigated the association between genetic variations of *MUC17* (A.K.A. *MUC3*) and endometriosis development based on the finding of gene expressing profiling in mucinous ovarian carcinoma (MOC), which likely arises from endometriosis [20, 21]. In this study, the mucin genes *MUC2*, *MUC3A*, and *MUC17* were found to be upregulated in MOC [22]. Similar to *MUC4*, *MUC17* encodes a membrane-bound mucin containing two EGF (epidermal growth factor)-like domains and a SEA (sea urchin sperm protein, enterokinase and agrin) domain in the extracellular portion, which can interact with human EGF receptor 2 (EGFR2) on the adjacent cell surface. Thus, the growth signal can be transduced to stimulate cellular proliferation [23]. Of note, MUC17 overexpression can also be found in colon and pancreatic cancers [24]. Although several lines of evidence support a potent role of MUC17 in tumor development, its role in endometriosis remains unclear. To investigate the possible link with endometriosis, we studied the association of *MUC17* genetic variants with the ovarian cancer biomarker CA125 and the related infertility.

## Methods

### Study population

One hundred eighty-nine Taiwanese patients who underwent surgery for benign ovarian disease and pathology-proven endometriosis at China Medical University Hospital from 1998 to 2011 were enrolled. These patients were diagnosed with ovarian cysts by ultrasound and confirmed by surgical operation. Symptoms of infertility and CA125 levels were also recorded for each patient during the study. Patients who failed to have pathology-proven endometriosis were excluded from this study. Another 191 healthy women were collected as controls who had regular health checkups at the same hospital. Ultrasound assessment indicated healthy conditions in the abdominal cavities of these controls. Both the controls and patients shared similar age profiles. This study was approved by the Institutional Review Board of China Medical University Hospital. Written informed consent was obtained from patients for scientific research and publication of this study.

### Genotyping

Genomic DNA was extracted from peripheral blood leukocytes according to the manufacturer’s protocol (Genomic DNA kit; Qiagen, Valencia, CA, USA). DNA fragments containing the target single-nucleotide polymorphism (SNP) sites were amplified by polymerase chain reaction (PCR) using the *Taqman* SNP Genotyping Assay System from Applied Biosystems, Inc. (Carlsbad, CA, USA). The probe search and design are available from the ABI SNP genotyping databank and are listed in Additional file 1. The PCR amplification conditions were as follows: 95 °C for 5 minutes, followed by 40 cycles at 95 °C for 10 seconds, 56 °C for 10 seconds and 72 °C for 20 seconds, with one additional cycle at 72 °C for 5 minutes. Genetic variations were detected by reading fluorescence signals of the PCR products. A positive signal indicates a perfect match between the probe and tested DNA, thus identifying the allele type.

### Statistical analysis

The allelic and genotypic frequency distributions for five SNP sites in endometriosis patients and controls were performed by *χ*^2^ analysis using *SPSS* software (version 10.0; SPSS Inc. Chicago, IL, USA). Allelic and genotypic frequencies were expressed as percentages of the total number of alleles and genotypes. Odds ratios (ORs) were calculated for the allelic and genotypic frequencies with 95 % confident intervals (95 % CIs). The major (wild-type) allele was used as the reference group. Lewontin's D' (|D'|) and the linkage disequilibrium (LD) coefficient *r*^2^ were determined between selected pairs of biallelic loci. Haploview version 3.2 software (Broad Institute, Cambridge, MA) was used to examine the structure of the LD block.

### Prediction of mRNA secondary structures adjacent to the SNP sites

The sequence of rs10953316, as well as the reference sequence, were submitted to the RNAstructure web server (http://rna.urmc.rochester.edu/RNAstructureWeb/index.html) [25], and structures with maximum expected accuracy were selected. Among the multiple algorithms in the RNAstructure web server, MaxExpect was utilized to generate structures composed of highly probable base pairs with the highest probability of being accurate.

### Immunohistochemistry

A total of 76 ectopic endometrial tissues were collected from patients with different genotypes at the rs10953316 SNP site. Rabbit anti-MUC17 polyclonal antibody (ab122184) was purchased from Abcam PLC., Cambridge, USA. Immunohistochemistry was performed with an antibody dilution of 1:50 using standard protocols with an EnVision + System peroxidase kit (DAKO Agilent Technologies, Glostrup, Denmark). Immunointensity of MUC17 in endometrial epithelium was independently scored by two pathologists and specific membrane staining was labeled as: weak positive, moderately positive, or strongly positive. For discordant cases, a third investigator was brought in to score and the final intensity score was determined by the majority scores.

## Results

### *MUC17* polymorphisms and endometriosis

**Fig. 1:**
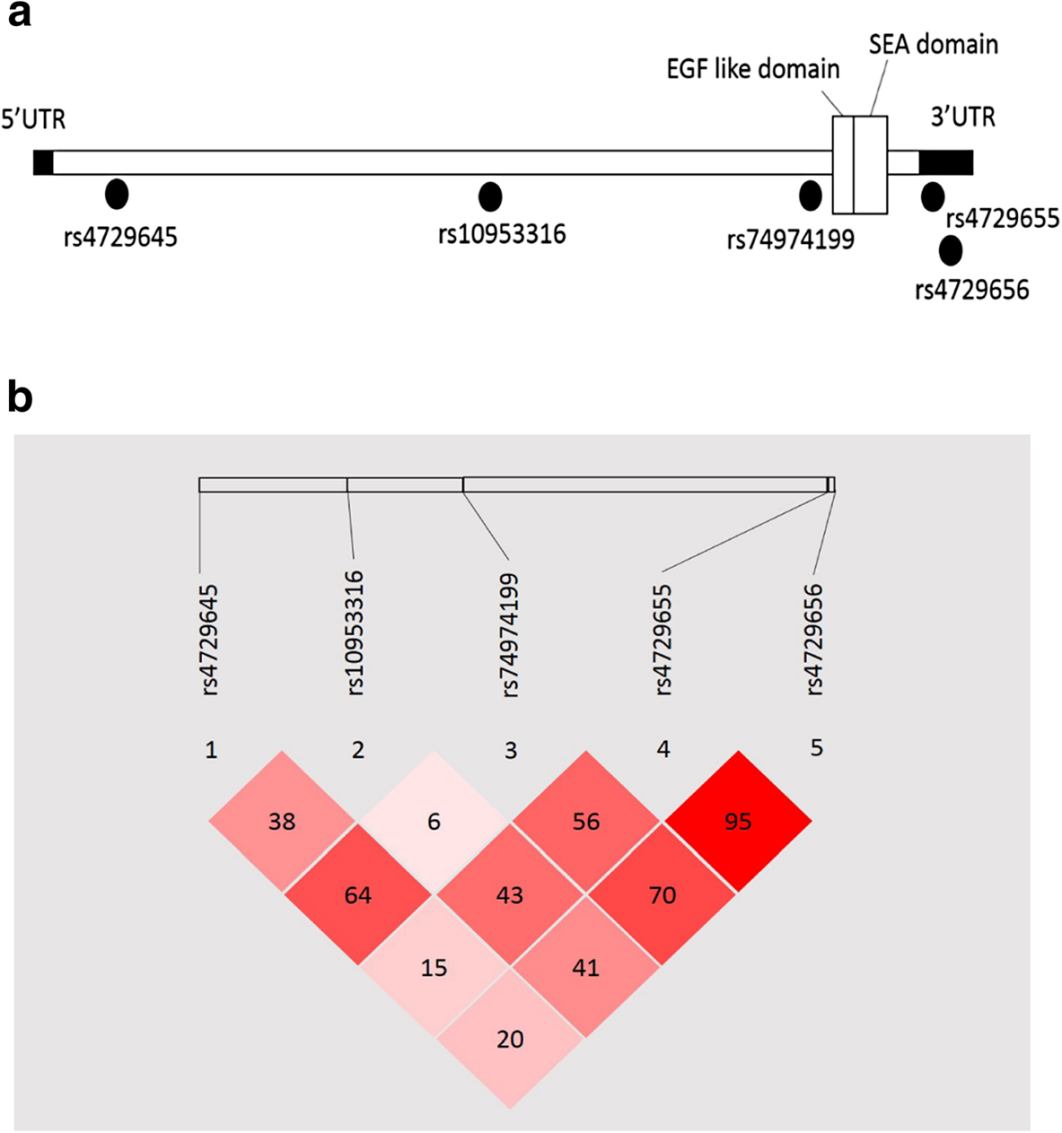
Locations of the selected SNPs and their linkage disequilibrium association in the *MUC17* gene in Taiwanese endometriosis patients. (**a**) Genetic alterations of rs4729645, rs10953316 and rs74974199 are located in the coding sequence region, while those of rs4729655 and rs4729656 are located at the 3′ end of the untranslated region in *MUC17*. (**b**) Values are shown with Lewontin’s D’

To examine whether SNPs in *MUC17* play a role in endometriosis, five SNPs were selected for this study, with a frequency greater than 5 % in the Chinese Han in Beijing from the International HapMap Project databank. The SNPs locations in *MUC17* are indicated in Fig. 1a. These SNPs were either located in the open reading frame region or the 3′-untranslated region (3′-UTR) of *MUC17*. The LD values of selected SNPs in patients are shown in Fig. 1B. Genotype analysis and allele distributions of SNPs in *MUC17* in the patients and controls are analyzed in Table 1. The major (wild-type) alleles were used as the references. Genetic analysis showed a significant association between endometriosis and genetic variation at the SNP site of rs10953316 with a lower frequency of the A allele in patients than in controls (*p* = 0.008; OR = 0.53; 95 % CI: 0.36-0.79) (Table 1). Genotype analysis of the rs10953316 site supported the protective potential of the AG genotype in endometriosis development compared with the reference GG genotype (OR = 0.45; 95 % CI: 0.29-0.7). The AA genotype was not found to show a dominant effect due to the limited sample size. Allelic and genotype analyses of the other SNPs showed no statistically significant differences.

**Table 1:** Genotype and allele distributions of SNPs in *MUC17* gene in Taiwanese endometriosis patients and controls

	*Genotype/allele*	*No. (%) of patients*	*No. (%) of controls*	*P-value* ^*a*^	*Corrected P-value* ^*b*^	*OR*	*95 % CI*
rs4729645	TT	0 (0.0)	2 (1.0)	0.13	0.65	0.00	NA
	CT	61 (33.0)	76 (39.8)			0.73	0.48-1.12
	CC	124 (67.0)	113 (59.2)			1.00	
	T	61 (16.5)	80 (20.9)	0.12	0.6	0.71	0.47-1.09
	C	309 (83.5)	302 (79.1)			1.00	
rs10953316	AA	1 (0.5)	1 (0.5)	0.0018*	0.009*	0.77	0.05-12.65
	AG	45 (23.8)	77 (40.5)			0.45	0.29-0.70
	GG	143 (75.7)	112 (58.9)			1.00	
	A	47 (12.3)	79 (20.8)	0.0016*	0.008*	0.53	0.36-0.79
	G	335 (87.7)	301 (79.2)			1.00	
rs74974199	CC	3 (1.6)	1 (0.5)	0.24	1.2	2.86	0.29-28.61
	CG	36 (19.4)	48 (25.4)			0.71	0.44-1.17
	GG	14 7 (79.0)	140 (74.1)			1.00	
	C	42 (11.3)	50 (13.2)	0.42	2.1	0.83	0.54-1.29
	G	330 (88.7)	328 (86.8)			1.00	
rs4729655	TT	27 (14.6)	21 (11.1)	0.58	2.90	1.35	0.71-2.54
	CT	96 (51.9)	104 (54.7)			0.97	0.62-1.51
	CC	62 (33.5)	65 (34.2)			1.00	
	T	150 (40.5)	146 (38.4)	0.55	2.75	1.09	0.82-1.46
	C	220 (59.5)	234 (61.6)			1.00	
rs4729656	TT	21 (11.3)	19 (10.0)	0.90	4.5	1.17	0.59-2.34
	AT	82 (44.1)	83 (43.7)			1.05	0.68-1.61
	AA	83 (44.6)	88 (46.3)			1.00	
	T	124 (33.3)	121 (31.8)	0.66	3.3	1.07	0.79-1.45
	A	248 (66.7)	259 (68.2)			1.00	

*Indicates statistical significance

^a^P-values were calculated using *χ*2 test

^b^P-values were corrected by Bonferroni correction

### *MUC17* polymorphisms and infertility

Because endometriosis is considered one of the factors that cause infertility, we next studied the relationship between *MUC17* SNPs and infertility. The endometriosis patients were sub-grouped into the fertile and infertile groups for allele distribution analysis. The results shown in Table 2 indicate that the genetic variant rs4729655 is a protective genetic factor for infertility in patients (*p* = 0.0465; OR = 0.42; 95 % CI: 0.21-0.82). The genetic variant at rs4729656 also showed a protective tendency, although this result did not reach statistical significance after Bonferroni correction.

**Table 2:** Allele distributions of SNPs in *MUC17* gene *vs.* infertility

*Polymorphism*	*Infertile*	*Non-Infertile*	*P-value* ^*a*^	*Corrected P-value* ^*b*^	*OR*	*95 % CI*
	*MAF*	*MAF*				
rs4729645	23.1	13.8	0.090	0.45	1.83	0.87-3.82
rs10953316	9.6	12.2	0.60	3.0	0.84	0.46-1.53
rs74974199	13.5	11.4	0.68	3.40	1.20	0.49-2.90
rs4729655	25.0	44.5	0.0093*	0.0465*	0.42	0.21-0.82
rs4729656	21.2	35.4	0.046*	0.23	0.48	0.24-0.98

*Indicates statistical significance

MAF: minor allele frequency

^a^P-values were calculated using *χ*2 test

^b^P-values were corrected by Bonferroni correction

**Table 3:** Allele distributions of SNPs in *MUC17* gene *vs.* CA-125 level

*Polymorphism*	*CA125 ≥ 35* ^*c*^	*CA125 < 35*	*P-value* ^*a*^	*Corrected P-value* ^*b*^	*OR*	*95 % CI*
	*MAF*	*MAF*				
rs4729645	15.1	13.6	0.76	3.80	1.12	0.61-2.13
rs10953316	13.0	12.5	0.91	4.55	1.03	0.42-2.51
rs74974199	14.4	5.7	0.045*	0.225	2.75	0.99-7.61
rs4729655	64.4	46.6	0.0076*	0.038*	2.08	1.20-3.57
rs4729656	28.8	44.3	0.015*	0.075	0.49	0.28-0.87

*Indicates statistical significance

MAF: minor allele frequency

^a^P-values were calculated using *χ*2 test

^b^P-values were corrected by Bonferroni correction

^c^CA125 level: KU/L

### *MUC17* polymorphism and CA125 levels

Cancer antigen 125 (CA125), also known as MUC16, is a commonly used serum biomarker to monitor the progress and recurrence of ovarian malignancy and benign conditions, including endometriosis. It has been shown that serum CA125 levels are markedly elevated in women with cystic ovarian endometriosis and/or deeply infiltrating endometriosis compared with women in the luteal phase with minimal or mild endometriosis [26]. Therefore, we asked whether SNPs in *MUC17* can affect the serum CA125 levels in patients. Patients were sub-grouped into two groups for genetic analyses based on the CA125 level: high CA125 (≥35 KU/L) and low CA125 (<35 KU/L) groups [27, 28]. Our data indicated that patients with the rs4729655 SNP showed a significantly high expression level of CA125 (*p* = 0.038; OR = 0.48; 95 % CI: 0.28-0.83). Genetic variation at rs74974199 also showed a correlation with elevated CA125 expression levels; however, such correlation did not reach statistical significance after Bonferroni correction. By contrast, patients with genetic variation at rs4729656 showed a tendency toward a lower CA125 level (Table 3).

### *MUC17* rs10953316 polymorphism controls its protein expression level

**Fig. 2:**
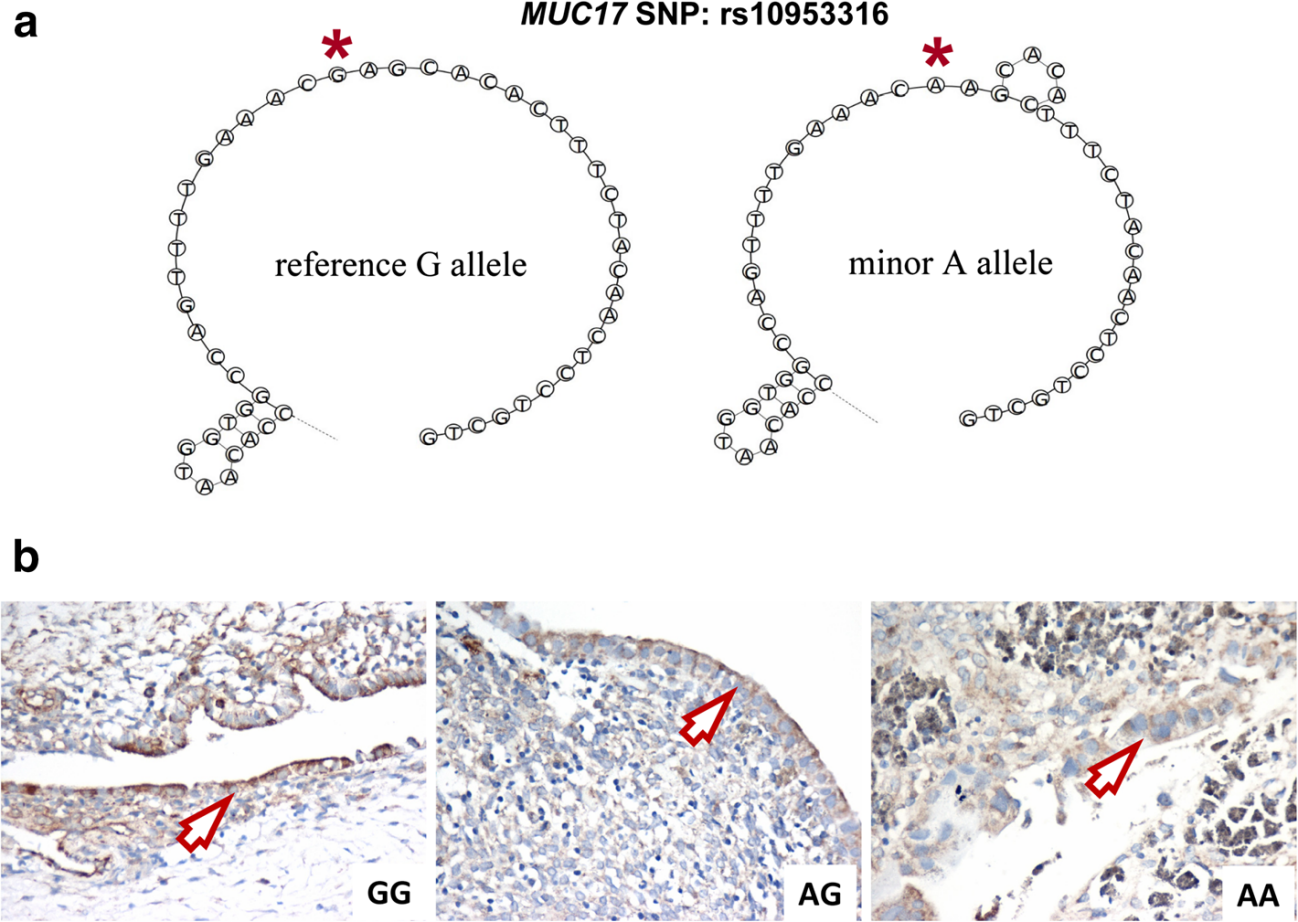
Influence of rs10953316 SNP on MUC17 mRNA structure and protein expression. (**a**) Partial mRNA structure of wild-type and rs10953316 SNP in *MUC17* were predicted by MaxExpect. Symbol *indicates the reference allele G and minor allele A sites of the rs10953316 SNP. (**b**) Immunohistochemistry against MUC17 was performed on endometrial tissues in patients. Representative staining results in patients with GG (*left*), AG (*middle*), and AA (*right*) genotypes at rs10953316 SNP site are shown. Specific staining can be detected at the edges of endometrium (arrows)

Based on above findings, the rs10953316 polymorphism was defined as a unique genetic factor regulating endometriosis development. However, this SNP is a silent genetic variation resulting in no amino acid change (Thr2355Thr). Therefore, we asked whether such a SNP can induce an RNA structural conformational change using the MaxExpect algorithm from the RNAstructure web server [25]. MaxExpect analysis revealed that the mRNA with genetic variation at rs10953316 forms an additional loop adjacent to the SNP site (Fig. 2a). Because mRNA structural change could be a hindrance to its stability and translation efficiency [29], we performed immunohistochemistry on endometriosis tissues to see whether such variation can influence the total amount of MUC17. As shown in Fig. 2B, patients with AA genotype at rs10953316 showed low MUC17 levels in their endometrium, patients with GA genotype showed moderate levels, and strong staining could be found in patients with GG genotype.

## Discussion

In this study, we showed molecular evidence that the A allele of rs10953316 in *MUC17* may function as a protective factor in endometriosis development. One of our limitations is the relatively small sample size, so that genotype AA at rs10953316 did not show a stronger effect (OR = 0.77, 95 % CI: 0.05-12.65) than the genotype AG (OR = 0.45; 95 % CI: 0.29-0.7). Although the rs10953316 polymorphism is a silent genetic variation, the total protein amount of MUC17 can be influenced probably due to RNA structural conformational change (Fig. 2). Our finding supports the previous hypothesis that structural change in RNA coding region can lead to ribosome pause and affect translational efficiency [29]. Similar finding was also evidenced by Nackley *et al.* that haplotypes divergent in synonymous changes in human *COMT* gene alter the mRNA structure with additional loops and reduced the amount of translated protein, which subsequently affected its function such as pain perception [30]. Because the extracellular domain of MUC17 protein contains several EGF-like functional domains, reduction of MUC17 expression by rs10953316 SNP may down-regulate the erbB signaling leading to inhibition of cell proliferation [23]. In addition, SEA domain at the C-terminal region of MUC17 is critical for its autoproteolysis, which controls cell migration and invasion [31]. These studies provide clues for us to presume that rs10953316 SNP plays a protective role in endometriosis development by reducing total MUC17 expression and the downstream signaling, whereas the detailed mechanism remains to be further investigated.

Another SNP, rs4729655, was linked to endometriosis-related infertility and was associated with abnormally high levels of CA125. The rs4729655 SNP site is located at the 3′-UTR of *MUC17*, a regulatory region for mRNA stability and miRNA binding. We next analyzed whether there was any possible miRNA targets gained or lost due to genetic variation at the rs4729655 SNP site. Our study found that genetic variation at rs4729655 resulted in the gain of miR-4508 and loss of miR-3158-3p toward the 3′-UTR of *MUC17*; thus, this SNP site may be involved in multiple miRNA interactions and gene regulation. Our previous study showed that a miR-125b binding site variation at the 3′-UTR of *BMPR1B* defines endometriosis susceptibility and CA125 levels [32]. Currently, biofunctions and downstream targets of miR-4508 and miR-3158-3p are still largely unknown. However, studies on the miRNAome of different cancer types using a deep sequencing approach provided solid evidence indicating the involvement of these two miRNAs in cancer development [33–36]. Particularly, miR-3158-3p expression was found to be associated with disease states in the female reproductive tract, including ovarian cancer and endometriosis [35]. Whether dysregulation of these two miRNAs could lead to infertility remains unclear. Further studies on these two miRNAs may provide insights to uncover the detailed mechanisms regarding how they regulate the development of female reproductive diseases.

One obstacle of this study was that the SNP associated with endometriosis development did not show an influence on infertility or CA125 levels in patients, and *vice versa*. Our data may reflect that more than 50 % of patients with endometriosis-related infertility are diagnosed as having minimal or mild clinical symptoms [37]. By contrast, the sensitivity and specificity of the CA125 blood test was also found to be limited, not necessary correlating with clinical stages. Therefore, it was recommended as an indication that women with high CA125 levels should receive further diagnostic screening and treatment [38, 39]. Cytokine/hormone networking, good oocyte quality and the success of embryo implantation may be the key players to determine endometriosis-related infertility. Because mucins have been found to play roles in regulating the immune responses of the reproductive tract and cellular proliferation of endometrial cells, whether MUC17 also plays such a roles is an interesting topic for us to further investigate.

## Conclusions

In this study, we analyzed and found the association between *MUC17* polymorphisms and endometriosis, endometriosis-related infertility and CA125 in a Taiwanese population. However, the relatively small sample size is one limitation of this study, and the conclusions should be further validated by another study cohort. The detailed regulatory mechanisms regarding how MUC17 functions and collaborates with its binding miRNAs in promoting endometriosis development remain to be elucidated.

## Additional file

Additional file 1: Table S1. Probes used for analyzing SNPs in *MUC17* gene. (PDF 108 kb)

## Abbreviations

CA125: cancer antigen 125
MOC: mucinous ovarian cancer
SNP: single-nucleotide polymorphism

## References

[1] Kinkel K, Frei KA, Balleyguier C, Chapron C (2006). Diagnosis of endometriosis with imaging: a review. European radiology.

[2] Jenkins S, Olive DL, Haney AF (1986). Endometriosis: pathogenetic implications of the anatomic distribution. Obstetrics and gynecology.

[3] Ballard K, Lowton K, Wright J (2006). What's the delay? A qualitative study of women's experiences of reaching a diagnosis of endometriosis. Fertility and sterility.

[4] Eskenazi B, Warner ML (1997). Epidemiology of endometriosis. Obstetrics and gynecology clinics of North America.

[5] Berkley KJ, Rapkin AJ, Papka RE (2005). The pains of endometriosis. Science.

[6] Van Gorp T, Amant F, Neven P, Vergote I, Moerman P (2004). Endometriosis and the development of malignant tumours of the pelvis. A review of literature. Best practice & research Clinical obstetrics & gynaecology.

[7] Orezzoli JP, Russell AH, Oliva E, Del Carmen MG, Eichhorn J, Fuller AF (2008). Prognostic implication of endometriosis in clear cell carcinoma of the ovary. Gynecologic oncology.

[8] Kobayashi H, Kajiwara H, Kanayama S, Yamada Y, Furukawa N, Noguchi T, Haruta S, Yoshida S, Sakata M, Sado T, Oi H (2009). Molecular pathogenesis of endometriosis-associated clear cell carcinoma of the ovary (review). Oncology reports.

[9] Nezhat F, Datta MS, Hanson V, Pejovic T, Nezhat C, Nezhat C (2008). The relationship of endometriosis and ovarian malignancy: a review. Fertility and sterility.

[10] Yonezawa S, Higashi M, Yamada N, Yokoyama S, Goto M (2010). Significance of mucin expression in pancreatobiliary neoplasms. Journal of hepato-biliary-pancreatic sciences.

[11] Groneberg DA, Peiser C, Dinh QT, Matthias J, Eynott PR, Heppt W, Carlstedt I, Witt C, Fischer A, Chung KF (2003). Distribution of respiratory mucin proteins in human nasal mucosa. The Laryngoscope.

[12] Filipe MI (1979). Mucins in the human gastrointestinal epithelium: a review. Investigative & cell pathology.

[13] Gipson IK, Ho SB, Spurr-Michaud SJ, Tisdale AS, Zhan Q, Torlakovic E, Pudney J, Anderson DJ, Toribara NW, Hill JA (1997). Mucin genes expressed by human female reproductive tract epithelia. Biology of reproduction.

[14] Gollub EG, Goswami S, Kouba D, Marom Z (1993). Regulation of mucin gene expression in secretory epithelial cells. Biochemical and biophysical research communications.

[15] Audie JP, Tetaert D, Pigny P, Buisine MP, Janin A, Aubert JP, Porchet N, Boersma A (1995). Mucin gene expression in the human endocervix. Human reproduction.

[16] Vlad AM, Diaconu I, Gantt KR (2006). MUC1 in endometriosis and ovarian cancer. Immunologic research.

[17] Nath S, Mukherjee P (2014). MUC1: a multifaceted oncoprotein with a key role in cancer progression. Trends in molecular medicine.

[18] Chang CY, Chang HW, Chen CM, Lin CY, Chen CP, Lai CH, Lin WY, Liu HP, Sheu JJ, Tsai FJ (2011). MUC4 gene polymorphisms associate with endometriosis development and endometriosis-related infertility. BMC medicine.

[19] Chang CY, Chen Y, Lin WC, Chen CM, Chen CP, Lee SC, Sheu JJ, Tsai FJ (2012). MUC2 polymorphisms are associated with endometriosis development and infertility: a case–control study. BMC medical genetics.

[20] Ko ML, Pan HS (2008). Identical twins with ovarian endometriosis and mucinous borderline tumor: an unusual association. Fertility and sterility.

[21] Lee KR, Nucci MR (2003). Ovarian mucinous and mixed epithelial carcinomas of mullerian (endocervical-like) type: a clinicopathologic analysis of four cases of an uncommon variant associated with endometriosis. International journal of gynecological pathology: official journal of the International Society of Gynecological Pathologists.

[22] Heinzelmann-Schwarz VA, Gardiner-Garden M, Henshall SM, Scurry JP, Scolyer RA, Smith AN, Bali A, Vanden Bergh P, Baron-Hay S, Scott C, Fink D, Hacker NF, Sutherland RL, O'Brien PM (2006). A distinct molecular profile associated with mucinous epithelial ovarian cancer. British journal of cancer.

[23] Bafna S, Kaur S, Batra SK (2010). Membrane-bound mucins: the mechanistic basis for alterations in the growth and survival of cancer cells. Oncogene.

[24] Hattrup CL, Gendler SJ (2008). Structure and function of the cell surface (tethered) mucins. Annual review of physiology.

[25] Reuter JS, Mathews DH (2010). RNAstructure: software for RNA secondary structure prediction and analysis. BMC bioinformatics.

[26] Muyldermans M, Cornillie FJ, Koninckx PR (1995). CA125 and endometriosis. Human reproduction update.

[27] Nossov V, Amneus M, Su F, Lang J, Janco JM, Reddy ST, Farias-Eisner R (2008). The early detection of ovarian cancer: from traditional methods to proteomics. Can we really do better than serum CA-125?. American journal of obstetrics and gynecology.

[28] Bagan P, Berna P, Assouad J, Hupertan V, Le Pimpec BF, Riquet M (2008). Value of cancer antigen 125 for diagnosis of pleural endometriosis in females with recurrent pneumothorax. The European respiratory journal.

[29] Mortimer SA, Kidwell MA, Doudna JA (2014). Insights into RNA structure and function from genome-wide studies. Nature reviews Genetics.

[30] Nackley AG, Shabalina SA, Tchivileva IE, Satterfield K, Korchynskyi O, Makarov SS, Maixner W, Diatchenko L (2006). Human catechol-O-methyltransferase haplotypes modulate protein expression by altering mRNA secondary structure. Science.

[31] Peng Z, He Y, Yang Y, Zhu R, Bai J, Li Y, Yu H, Zhang X, Chen L, Chen W, Fang D, Wang R (2010). Autoproteolysis of the SEA module of rMuc3 C-terminal domain modulates its functional composition. Archives of biochemistry and biophysics.

[32] Chang CY, Chen Y, Lai MT, Chang HW, Cheng J, Chan C, Chen CM, Lee SC, Lin YJ, Wan L, Tsai PW, Yang SH, Chung C, Sheu JJ, Tsai FJ (2013). BMPR1B up-regulation via a miRNA binding site variation defines endometriosis susceptibility and CA125 levels. PloS one.

[33] Jima DD, Zhang J, Jacobs C, Richards KL, Dunphy CH, Choi WW, Au WY, Srivastava G, Czader MB, Rizzieri DA, Lagoo AS, Lugar PL, Mann KP, Flowers CR, Bernal-Mizrachi L, Naresh KN, Evens AM, Gordon LI, Luftig M, Friedman DR, Weinberg JB, Thompson MA, Gill JI, Liu Q, How T, Grubor V, Gao Y, Patel A, Wu H, Zhu J, Blobe GC, Lipsky PE, Chadburn A, Dave SS (2010). Deep sequencing of the small RNA transcriptome of normal and malignant human B cells identifies hundreds of novel microRNAs. Blood.

[34] Stark MS, Tyagi S, Nancarrow DJ, Boyle GM, Cook AL, Whiteman DC, Parsons PG, Schmidt C, Sturm RA, Hayward NK (2010). Characterization of the Melanoma miRNAome by Deep Sequencing. PloS one.

[35] Creighton CJ, Benham AL, Zhu HF, Khan MF, Reid JG, Nagaraja AK, Fountain MD, Dziadek O, Han D, Ma L, Kim J, Hawkins SM, Anderson ML, Matzuk MM, Gunaratne PH (2010). Discovery of Novel MicroRNAs in Female Reproductive Tract Using Next Generation Sequencing. PloS one.

[36] Persson H, Kvist A, Rego N, Staaf J, Vallon-Christersson J, Luts L, Loman N, Jonsson G, Naya H, Hoglund M, Borg A, Rovira C (2011). Identification of New MicroRNAs in Paired Normal and Tumor Breast Tissue Suggests a Dual Role for the ERBB2/Her2 Gene. Cancer Res.

[37] Holoch KJ, Lessey BA (2010). Endometriosis and Infertility. Clin Obstet Gynecol.

[38] Baron JA (2012). Screening for cancer with molecular markers: progress comes with potential problems. Nat Rev Cancer.

[39] Fritsche HA, Bast RC (1998). CA 125 in ovarian cancer: Advances and controversy. Clin Chem.

