# Supplementary material for: Genetic variations of MUC17 are associated with endometriosis development and related infertility
Source: BMC Med Genet. 2015 Aug 19;16:60. doi: 10.1186/s12881-015-0209-7 (PMC4593232; doi:10.1186/s12881-015-0209-7)
Supplement: Additional file 1: Table S1. — Probes used for analyzing SNPs in MUC17 gene. (PDF 108 kb) [file 12881_2015_209_MOESM1_ESM.pdf]

Additional file 1. Probes used for analyzing SNPs in *MUC17* gene

dbSNP rs#	Allele frequency*		Amino acid change	ABI probe ID
rs4729645	C:82%	T:18%	Thr339Met	AHAA0I0[b]
rs10953316	A:18%	G:82%	Thr2355Thr	AH6RM4Q[b]
rs74974199	C:24%	G:76%	Gly3932Arg	AHBJYO8[b]
rs4729655	T:36%	C:64%	NA[a]	C__27935224_10
rs4729656	T:32%	A:68%	NA[a]	C__27978557_20

*Allele frequencies listed are from Han Chinese population distributions in HapMap database

[a] SNP within 3'-UTR region of *MUC17*

[b] Custom designed probe
